# Orexin, orexin receptor antagonists and central cardiovascular control

**DOI:** 10.3389/fnins.2013.00257

**Published:** 2013-12-30

**Authors:** Pascal Carrive

**Affiliations:** Blood Pressure, Brain and Behavior Laboratory, School of Medical Sciences, University of New South WalesSydney, NSW, Australia

**Keywords:** Ox1R, Ox2R, blood pressure, heart rate, sympathetic, rostral ventrolateral medulla, psychological stress, SHR

## Abstract

Orexin makes an important contribution to the regulation of cardiovascular function. When injected centrally under anesthesia, orexin increases blood pressure, heart rate and sympathetic nerve activity. This is consistent with the location of orexin neurons in the hypothalamus and the distribution of orexin terminals in the central autonomic network. Thus, the two orexin receptors, Ox1R and Ox2R, which have partly overlapping distributions in the brain, are expressed in the sympathetic preganglionic neurons (SPN) of the thoracic cord as well as in regions such as the pressor area of the rostral ventrolateral medulla (RVLM). Both Ox1R and Ox2R appear to contribute to the cardiovascular effects of orexin, although Ox1R is probably more important. Blockade of orexin receptors reduces the cardiovascular response to certain stressors, especially psychogenic stressors such as novelty, aggressive conspecifics and induced panic. Blockade of orexin receptors also reduces basal blood pressure and heart rate in spontaneous hypertensive rats, a model of essential hypertension. Thus, there is a link between psychogenic stress, orexin and elevated blood pressure. The use of dual orexin receptor antagonists (DORAs) and selective orexin receptor antagonists (SORAs) may be beneficial in the treatment of certain forms of hypertension.

## Introduction

Orexin contributes to the central regulation of cardiovascular function because it is a key player in the control of wakefulness and arousal. Indeed, to stay awake and interacting with the environment requires autonomic and cardiovascular adjustments not only to support muscle activity but also to prepare for muscle activity, as is the case for motivated behaviors and emotions. This link with arousal and motivated behavior makes orexin a significant new player in the field of central cardiovascular control. Most of our knowledge of central cardiovascular control is based on brainstem-mediated short-term homeostatic regulation that has been studied in the anesthetized preparation, while comparatively little is known about suprabulbar regulation in relation to behavior and emotions. In other words, orexin reveals another dimension of central cardiovascular control, one that could lead to new therapeutic interventions in some forms of cardiovascular diseases, such as for example, stress related hypertension or essential hypertension.

## Early studies

The cardiovascular effects of orexin were first demonstrated in 1999 by Samson et al. (1999) and Shirasaka et al. (1999). Both studies showed that orexin A and orexin B (OxA, OxB, 1–5 nmol) could evoke marked and sustained increases in blood pressure when injected in the lateral ventricle of freely moving rats. Both reported a stronger effect with OxA than OxB. Most importantly, Shirasaka et al. showed (i) that this pressor effect was associated with an increase in heart rate and renal sympathetic nerve activity and (ii) that the same effects could still be evoked under anesthesia. The later two findings established without doubt that the cardiovascular effect of orexin was due to a direct central sympathoexcitatory action. This was confirmed shortly after in a series of three papers by Dun and collaborators (Chen et al., 2000; Dun et al., 2000; Antunes et al., 2001). They showed that the same cardiovascular effects were still evoked (i) by intracisternal and intrathecal (T2–T3) injections of OxA and OxB and (ii) by microinjections of OxA in the vasopressor area of the rostral ventrolateral medulla [RVLM, (Chen et al., 2000; Dun et al., 2000)]. They also demonstrated that OxA and OxB directly depolarize vasopressor neurons of the RVLM and sympathetic preganglionic neurons (SPN) in the thoracic cord (Dun et al., 2000; Antunes et al., 2001).

Pharmacological blockade studies were not possible at the time due to lack of receptor antagonists. However, a seminal study by Kayaba et al. (2003) showed that orexin knock out mice had (i) a reduced basal blood pressure and (ii) a reduced cardiovascular response to a psychosocial stressor (resident-intruder test), but not to a noxious stimulus (tail pinch). This study suggested an important role of orexin in the cardiovascular response to motivated behavior.

## Anatomy of the orexin system in relation to the central autonomic network

### Orexin neurons and their connections

The neurons that make orexin are found in the dorsal part of the tuberal hypothalamus, nowhere else in the brain. The group is centered on the perifornical area (PeF) and extends medially into the dorsomedial hypothalamic nucleus and laterally into the lateral hypothalamic area (Peyron et al., 1998; Nambu et al., 1999). Interestingly, this region corresponds relatively well to the classic hypothalamic defense area, a region identified more than 50 years ago and from which powerful behavioral and cardiovascular responses can be evoked (Hilton, 1982; Carrive, 2011).

Specific inputs to orexin neurons, identified with a genetically encoded retrograde tracer (Sakurai et al., 2005) or from appositions of anterogradely labeled terminals (Yoshida et al., 2006), originate mostly from forebrain areas, either limbic [lateral septum, bed nucleus of the stria terminalis (BNST), amygdala, infralimbic cortex] or hypothalamic (preoptic area, posterior hypothalamus). These regions are also well known for their role in emotions and autonomic control (Saper, 2004).

**Figure 1:**
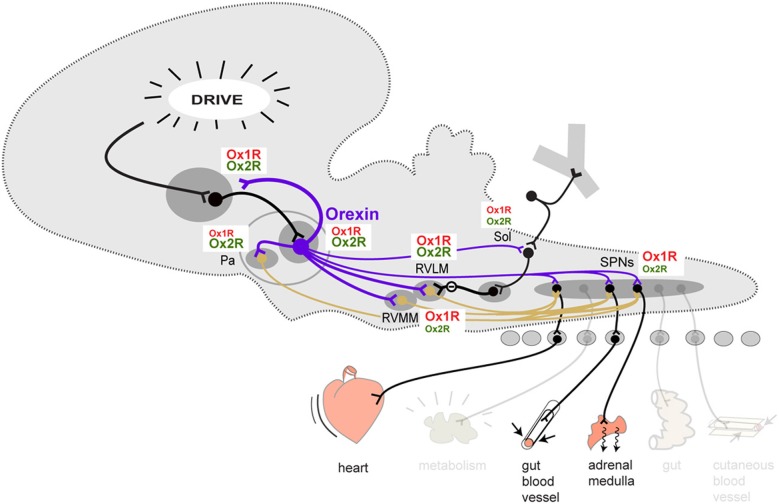
**Schematic overview of the orexinergic pathways involved in the descending control of sympathetic output to cardiovascular effectors**. The contribution of Ox1R and Ox2R at each level is represented by the relative size of the Ox1R and Ox2R labels. This is a tentative representation only, reflecting the current stage of our knowledge. Abbreviations: Pa, paraventricular nucleus of the hypothalamus; RVLM, rostral ventrolateral medulla; RVMM, rostral ventromedial medulla (plus medullary raphe); Sol, solitary nucleus; SPN, sympathetic preganglionic neurons.

On the output side, orexin terminals can be seen not only in the limbic structures described above where they make reciprocal connections, but also in all the autonomic centers of the hypothalamus and brainstem, including the periaqueductal gray (PAG), parabrachial nucleus, nucleus of the solitary tract (Sol), the premotor sympathetic centers of the paraventricular nucleus of the hypothalamus (Pa), rostral ventrolateral and ventromedial medulla (RVLM, RVMM) and medullary raphe (Figure 1) (Peyron et al., 1998; Nambu et al., 1999; Baldo et al., 2003; Ciriello and De Oliveira, 2003; Ciriello et al., 2003; Zheng et al., 2005; Shahid et al., 2012). Orexin neurons are also themselves premotor sympathetic neurons since they directly innervate SPN (Van Den Pol, 1999; Date et al., 2000; Llewellyn-Smith et al., 2003). Projections are also found in the dorsal motor nucleus of the vagus although the projection may be weak (Peyron et al., 1998; De Oliveira et al., 2003). Thus, orexin neurons can act at all levels of the central autonomic network, from limbic structures to premotor autonomic centers to the SPNs themselves.

### Orexin receptors and their distribution

There are two orexin receptors, Ox1R and Ox2R (Gotter et al., 2012). OxA can act on both Ox1R and Ox2R while OxB acts primarily on Ox2R. The two receptors have a differential, partly overlapping distribution in the brain, including within the central autonomic network (Figure 1). However, it is not clear how the two receptors relate to the cardiovascular functions of orexin.

Three main *in situ* hybridization studies have compared the distribution of the two receptors in the brain and hypothalamus (Trivedi et al., 1998; Lu et al., 2000; Marcus et al., 2001). They show that the BNST expresses both receptors while the amygdala primarily expresses Ox1R and the septum primarily Ox2R. In the hypothalamus, most areas express both receptors, except Pa, which appears to exclusively express Ox2R. In the brainstem, most of the autonomic areas described above also express both receptors, except for the A5 catecholaminergic cell group, which like the A6 group of the locus coeruleus exclusively expresses Ox1R. Single cell RT-PCR in SPNs show that Ox1R is easier to detect than Ox2R (Van Den Top et al., 2003).

Immunohistochemical studies of Ox1R and Ox2R distribution (Hervieu et al., 2001; Cluderay et al., 2002) have confirmed the overall distribution of the two receptors, but they also show more overlap than suggested by the *in situ* hybridization studies. For instance, the amygdala also contains Ox2R and the septum Ox1R. More importantly, Pa also contains a significant amount of Ox1R in both its magno- and parvocellular areas, which colocalizes with vasopressin/oxytocin and CRF, respectively (Hervieu et al., 2001; Bäckberg et al., 2002). Interestingly, orexin neurons themselves have both receptors as autoreceptors (Bäckberg et al., 2002; Yamanaka et al., 2010). Other reports confirm dense Ox1R labeling in the RVMM area (Ciriello and De Oliveira, 2003; Ciriello et al., 2003) and both Ox1R and Ox2R in C1 neurons of the RVLM (Shahid et al., 2012). The presence of Ox1R and Ox2R in SPNs remains to be verified by immunohistochemistry.

## Orexin receptors mediating the cardiovascular effects of centrally injected exogenous orexin

Given the partly overlapping distribution of Ox1R and Ox2R in central autonomic structures, it is not clear if one receptor or the other or both mediate(s) the cardiovascular effect of orexin. This question can be answered by challenging the effect of exogenous OxA (which acts on both receptors) with selective orexin receptor antagonists (SORA) or by using selective agonists. The two Ox1R SORA are SB334867, the most popular, and SB408124 (Scammell and Winrow, 2011; Gotter et al., 2012; Morairty et al., 2012). So far the only Ox2R SORA to have been used in this field of research is TCS-OX229 (Hirose et al., 2003), although a number of studies have also used the selective Ox2R agonist, [Ala11, D-Leu15]-OxB (Asahi et al., 2003).

### Orexin microinjected in the ventricles or subarachnoid space

Hirota et al. (2003) were the first to demonstrate that prior blockade of Ox1R with intracerebroventricular (icv) injections of the Ox1R antagonist SB334867 (50 nmol) could almost completely block the cardiovascular response of icv OxA (50 nmol). This was confirmed in the awake rat using smaller doses of icv OxA (3 nmol) and another Ox1R antagonist, SB408124 (3 nmol) (Samson et al., 2007). Similarly, spinal intrathecal injections of SB334867 (200 nmol) almost completely blocked the pressor, tachycardic, and sympathetic nerve response to intrathecal OxA (50 nmol) (Shahid et al., 2011). However, Huang et al. (2010) showed that the Ox2R antagonist TCS-OX229 (3 and 10 nmol) was more potent than SB334867 (3 and 10 nmol) in reducing the pressor and tachycardic effect of OxA (3 nmol) when injected in the cisterna magna. These studies suggest that Ox1R is important, but Ox2R, which has not been challenged as much as Ox1R, might be equally as important.

### Orexin microinjected within regions of the central autonomic network

#### SPN

Injection of OxA and OxB on identified SPN evokes strong depolarization due to a direct postsynaptic action (Antunes et al., 2001; Van Den Top et al., 2003). The response was as strong with OxA as with OxB, and OxA's effect was only reduced by 60% after blockade with SB334867 (Van Den Top et al., 2003). It led these authors to suggest that both receptors might be involved.

#### RVLM

A similar study on neonate RVLM pressor neurons by Huang et al. (2010) revealed equipotent depolarizing effect of OxA, OxB and the specific OxB agonist [Ala11, D-Leu15]-OxB. These authors further showed that TCS-OX229 was more potent than SB334867 in reducing the effect of OxA (Huang et al., 2010). Similar observations were reported by Shahid et al. (2012) who showed that OxA and [Ala11, D-Leu15]-OxB had equipotent cardiovascular effects when injected in the RVLM and that SB334867 reduced OxA's effect by half.

#### Sol

Injections of low doses (2.5–5 pmol) of OxA and OxB in the nucleus of the solitary nucleus evokes equipotent bradycradic and depressor responses (De Oliveira et al., 2003; Shih and Chuang, 2007; Ciriello et al., 2013), however at higher dose (>40 pmol) both evoke tachycardic and pressor responses, with OxA more potent than OxB. The effects appear to be mediated by both Ox1R and Ox2R (Shih and Chuang, 2007).

#### Others

When injected in the medullary raphe area, OxA produces tachycardic effects at low dose (2.5 pmol) and pressor and tachycardic effects at high dose (30 pmol) (Luong and Carrive, 2012). In contrast, OxA evokes bradycardic effects when injected in the external part of the Ambiguus nucleus, presumably by activating vagal preganglionic neurons (Ciriello and De Oliveira, 2003). However, it is not known what receptor mediates these effects, although these two areas have been shown to contain Ox1R.

To summarize, the cardiovascular effect of exogenous orexin can in large part be explained by an action through Ox1R at almost all levels (Figure 1). However, in the RVLM, one of the most important vasopressor center in the brain, an action through Ox2R appears as important, ifnot more. Ox2R may also contribute at the level of SPNs themselves.

## Orexin receptors mediating the cardiovascular responses of centrally released, endogenous orexin

Orexin microinjections may reveal important properties of orexin's loci and mode of action, but the main question is how endogenous orexin acts in the behaving animal, where and through which receptors. Coordinated release of orexin at multiple levels of the neuraxis in synchrony with activation of other non-orexinergic system would have far more subtle effects than those of a ventricular or intracerebral injection. The approach therefore consists in first identifying behavioral conditions or pathological states associated with orexin release and then challenging these responses or states with systemic injections of dual and selective orexin receptor antagonists (DORA and SORA). Almorexant (Brisbare-Roch et al., 2007; Morairty et al., 2012) is the main DORA that has been used so far in relation to central autonomic control. The SORAs are the same as those described above.

### Centrally evoked responses

Disinhibition of the PeF is one way of inducing an orexin-mediated response. Remarkably, although orexin neurons (i) contain other peptides and use glutamate as their main neurotransmitter and (ii) only represent a fraction of the output neurons of the perifornical area, still, 50% of the tachycardic and pressor response obtained by bicuculline injection in the perifornical hypothalamus is blocked by systemic administration of Almorexant (15 mg/kg, iv) (Iigaya et al., 2012). This indicates that the peptide plays an important role in the output of this area. As suggested by the cardiovascular effects of OxA in the medulla and spinal cord described above, it is likely that Almorexant will exert its effect via an action on the targets of orexin neurons, however, part of its action maybe in the perifornical area itself since Almorexant microinjections in the PeF can reduce the tachycardic and pressor responses to perifornical disinhibition (Stettner and Kubin, 2013).

Similar results have been reported with Ox1R SORAs. In the rabbit, SB334867 and SB408124 (7 mg/kg, iv) markedly reduced the pressor response evoked by electrical stimulation of the dorsal hypothalamus and PAG (Nisimaru et al., 2013). In conscious rat SB334867 (10 mg/kg, iv) also reduced by about 40–50% the tachycardic, pressor and hyperthermic response to muscimol injection in the medial preoptic area, an area that exerts a tonic inhibition of the dorsal hypothalamic area (Rusyniak et al., 2011).

### Psychological stressors and induced panic

A number of psychological stressors have been challenged with Almorexant or Ox1R SORAs. Novelty, conditioned fear to context, restraint and cold exposure, all evoke tachycardic and pressor responses, however, Almorexant (300 mg/kg, io) affected only novelty and contextual fear (Furlong et al., 2009). The pressor responses to novelty and conditioned fear, the tachycardic response to novelty and the cardiac sympathetic response to conditioned fear were reduced by 45% or more, but restraint and cold exposure were not significantly affected by Almorexant (Furlong et al., 2009). This led the authors to suggest that the stress responses to which orexin contribute might be more psychological than physical, reminiscent of the observation by Kayaba et al. (2003), that the cardiovascular response to pinch was not affected in knock out orexin mice, whereas that to social stress was.

The other studies have used Ox1R SORAs, mainly SB334867. A major study by Johnson et al. (2009) showed that both the pressor and tachycardic responses evoked by sodium lactate in panic prone rats were markedly reduced by systemic SB334867 (30 mg/kg, ip). A similar effect was observed with SB408124 (30 mg/kg, ip also). The same high dose of SB334867 also reduced the pressor (but not bradycardic) response to hypercapnia (20% CO2, a suffocation signal) as well as the tachycardic (but not pressor) response to the anxiogenic partial inverse benzodiazepine agonist FG7142 (Johnson et al., 2012a,b). SB334867 (10 mg/kg, iv) also reduces the pressor but not tachycardic response to a moderate dose of metamphetamine (Rusyniak et al., 2012). Finally, a recent study with a new selective Ox1R antagonist (ACT335827, 100–300 mg/kg, io) reported a significant reduction of the tachycardic (but not pressor) response to social stress (Steiner et al., 2013).

This indicates that orexin contributes to the cardiovascular components of some forms of stress as initially shown in orexin knock-out mice by Kayaba et al. (2003). The Ox1R appears to play an important part in this, however the dose of Ox1R antagonists used in these studies is very high. They could be acting on Ox2R as well. Unfortunately, the contribution of Ox2R is not yet known as no study has tried to challenge these responses with Ox2R antagonists.

### Chronic stress-induced and spontaneous hypertension

Recent work has revealed a potentially important role of orexin in stress-induced or spontaneous forms of hypertension.

Xiao et al. (2012) used electric shocks over a period of 14 days to produce a stress-induced hypertensive state (+30 mmHg). Remarkably, this treatment doubled the number of orexin neurons and almost doubled the amount of Ox1R in the RVLM (Ox2R was not investigated). Consistent with this effect, unilateral microinjections of OxA in RVLM produced greater pressor and tachycardic responses in these animals. Conversely, RVLM injections of the Ox1R SORA SB408124 reduced systolic pressure and heart rate in those hypertensive animals but not in the normotensive non-stressed controls. Interestingly, Ox2R was also involved since the Ox2R SORA TCS-OX229 reduced systolic pressure in these hypertensive animals. This study shows that chronic stress can upregulate the orexin system and that both receptors, with possibly a dominance of the Ox1R, mediate the resulting hypertension. Most remarkable is the increase in orexin neurons as a result of chronic stress.

Finally, two recent studies suggest that essential hypertension in the Spontaneously hypertensive rat (SHR) may in part be due to an overactive orexin system. Thus, Li et al. (2013) have shown that oral administration of Almorexant in the conscious SHR reduces mean arterial blood pressure (~30 mmHg), heart rate and plasma noradrenaline during both wakefulness and NREM sleep, but has no significant effect in normotensive WKY rats. Lee et al. (2013) further showed in the anaesthetized SHR that icv TCS-OX229 (10 and 30 nmol) but not SB334867 (100 nmol) reduced MAP and HR. Surprisingly, there was a reduction of Ox2R density in the RVLM, but no change in PVN, DMH/PeF, and caudal NTS. Ox1R was also the same as in WKY in all four regions. Finally, bilateral injections of TCS-OX229 in the RVLM markedly lowered MAP (~30 mmHg), suggesting that the cause of the hypertensive phenotype in these animals could be an over-activation of Ox2R.

## Basal blood pressure and heart rate in orexin deficient humans and mice

If orexin upregulation leads to hypertension, then one would expect orexin downregulation or deficiency to lead to hypotension. Indeed, a reduced baseline blood pressure during wakefulness has been reported in orexin knock-out and orexin neuron-ablated mice (orexin-ataxin3 transgene) (Kayaba et al., 2003; Lo Martire et al., 2012). However, other studies have found no difference between these mice and wild type controls (Bastianini et al., 2011; Silvani et al., 2013). Interestingly, in human, patients with narcolepsy with cataplexy, which have reduced levels of orexin and orexin neurons, also have normal basal blood pressure when awake (Grimaldi et al., 2010, 2012; Dauvilliers et al., 2012). In contrast, and somewhat unexpectedly, baseline blood pressure during sleep tends to be higher than in controls. Thus, in both patients and transgenic mice, the circadian variation in blood pressure is consistently reduced. The dip in blood pressure that is normally seen between wakefulness and sleep is reduced, as is the rise in blood pressure when waking up from sleep (Bastianini et al., 2011; Dauvilliers et al., 2012; Grimaldi et al., 2012; Lo Martire et al., 2012; Silvani et al., 2013).

In terms of heart rate, the same studies in mice report either no change (Kayaba et al., 2003; Lo Martire et al., 2012) or an increase (Bastianini et al., 2011; Silvani et al., 2013) during wakefulness, whereas in patients both increases (Grimaldi et al., 2010, 2012; Sorensen et al., 2013) and decreases have been reported (Dauvilliers et al., 2012). During sleep, heart rate may be higher (Bastianini et al., 2011) or the same (Bastianini et al., 2011; Lo Martire et al., 2012; Silvani et al., 2013) in transgenic mice, and higher (Grimaldi et al., 2012) or the same (Dauvilliers et al., 2012) in patients. Nevertheless, as observed with blood pressure, the rise in heart rate at the transition between sleep and arousal is reduced in both transgenic mice and narcoleptic patients (Silvani et al., 2013; Sorensen et al., 2013).

To summarize, a chronic lack of orexin does not necessarily lead to a lower blood pressure and heart rate, but it will result in a blunted circadian variation of these parameters. There may be a simple explanation to this. As suggested by Grimaldi et al. (2012), the altered sleep/wake regulation could have a confounding effect opposing the direct sympatholytic effect of orexin deficiency. Simply put, trying not to fall asleep during the active period or conversely, being regularly awaken during the inactive period, will both increase sympathetic activity. Nevertheless, a more relevant observation with respect to narcoleptic patients is their reduced autonomic response to emotional stimuli, especially aversive ones (Tucci et al., 2003). In contrast, their cardiovascular response to basic homeostatic challenges such as head-up tilt, Valsalva maneuver and cold pressor test are unaffected (Grimaldi et al., 2010), which is consistent with orexin's primary role in motivated behavior.

## Conclusion

The orexinergic system plays an important role in central cardiovascular control. It is excitatory and contributes to the sympathetic response associated with motivated behavior, including some forms of stress. However, the mode of action of orexin is far from clear. Orexin terminals and orexin receptors are found in all the regions known to regulate cardiovascular function from sympathetic motor to premotor autonomic to limbic levels. So far anatomical and pharmacological evidences point toward a primary role for Ox1R, however this view is biased by the fact that most studies have used Ox1R SORAs (i.e., SB334867). Ox2R is also found in most central cardiovascular centers and when challenged is often found to be as important as Ox1R. Thus, it is not clear how the two receptors interact and if their effects are additive or synergistic. Nevertheless, blockade of orexin receptors can reduce the hypertension that is evoked by some acute psychological stressors, induced by chronic stress or simply spontaneous as in the case of adult SHR, a model of essential hypertension. Clearly there is an interesting link between psychogenic stress, orexin, and elevated blood pressure. Further research in SORAs and DORAs may well lead to the development of new anti-hypertensive drugs.

### Conflict of interest statement

The author declares that the research was conducted in the absence of any commercial or financial relationships that could be construed as a potential conflict of interest.
